# Sickle Cell Vaso-Occlusive Crisis Leading to Uteroplacental Malperfusion: A Case Report

**DOI:** 10.1055/a-2849-8204

**Published:** 2026-04-29

**Authors:** Annika van Oosbree, Gretchen Hahn, Sarah Shea

**Affiliations:** 1 2345 Department of Obstetrics/Gynecology, Medical University of South Carolina, Charleston, United States

**Keywords:** sickle cell disease, fetal demise, uterine hypoperfusion, neonatal morbidity

## Abstract

**Background** Sickle cell disease (SCD) in pregnancy is associated with substantially increased risks of stillbirth and neonatal death due to vaso-occlusive crises, chronic anemia, and impaired placental perfusion.

**Case** We present the case of a 25-year-old primigravida with HbSS disease who presented at 29 weeks’ gestation with a vaso-occlusive pain crisis. Although she was initially clinically stable with reassuring fetal testing, she developed acute fetal distress on hospital day 3, prompting an emergent cesarean delivery. Intraoperatively, the uterus appeared profoundly hypoperfused with minimal bleeding despite uterine atony. The neonate was delivered in asystole and required prolonged resuscitation. Postresuscitation examination raised concern for severe hypoxic ischemic encephalopathy, and the parents elected to transition to comfort care. The neonate died shortly after withdrawal of life-sustaining interventions. Placental pathology demonstrated prominent sickled erythrocytes and increased fetal nucleated red blood cells, consistent with prolonged impaired oxygen delivery.

**Conclusion** This case highlights the unpredictable and acute nature of fetal compromise in pregnancies complicated by SCD, which may occur prior to guideline-recommended antenatal surveillance. Although prophylactic transfusion remains controversial, emerging evidence suggests a potential benefit in selecting high-risk patients. Early multidisciplinary management, individualized transfusion strategies, and heightened vigilance for sudden fetal decompensation are essential to improving perinatal outcomes in SCD.

## Introduction

Sickle cell disease (SCD) in pregnancy is associated with significant maternal and perinatal morbidity and mortality, including markedly increased rates of stillbirth and neonatal death. The current evidence suggests that stillbirth rates are 2–12 times higher and neonatal death rates are two to five times higher in pregnancies affected by SCD when compared to the general population, 1 2 3 4 with the highest risks observed in those with HbSS disease. 1 These adverse outcomes reflect the combined effects of maternal vaso-occlusive crises, chronic anemia, and impaired placental perfusion.

We present the case of a 25-year-old primigravida with HbSS disease who presented at 29 weeks of gestation with a vaso-occlusive pain crisis. Her course was complicated by fetal distress, profound neonatal hypoxic ischemic injury secondary to impaired uteroplacental perfusion, and ultimate neonatal demise, underscoring the persistent challenges in optimizing maternal and perinatal outcomes in SCD-affected pregnancies and raising the question of the threshold for transfusion in these cases.

## Case Presentation

A 25-year-old G1P0 female at 29 ^1/7^ weeks of gestational age presented to labor and delivery for a sickle cell pain crisis. She was an established patient at an outpatient SCD specialty clinic, and she was prescribed a home pain regimen of scheduled hydroxyurea and acetaminophen–codeine as needed. Her medical history was otherwise significant for mild intermittent asthma, for which she used albuterol as needed. She had not previously been admitted for a sickle cell pain crisis during this pregnancy but had previously been admitted over a year prior for acute chest syndrome. She presented to the emergency department with severe generalized pain. Her admission labs were significant for a reticulocyte count of 325, a white blood cell count of 20,000 with a left shift, and a hemoglobin level of 7.7 g/dL, which was close to her baseline hemoglobin of 8.2 g/dL. A chest X-ray was negative for evidence of infection or acute chest syndrome. She required multiple doses of morphine without improvement in her pain, and the antepartum service was consulted for admission for pain management.

**Fig. 1:**
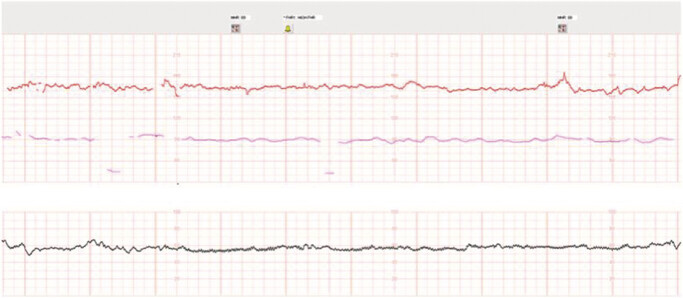
Admission nonstress test.

**Fig. 2:**
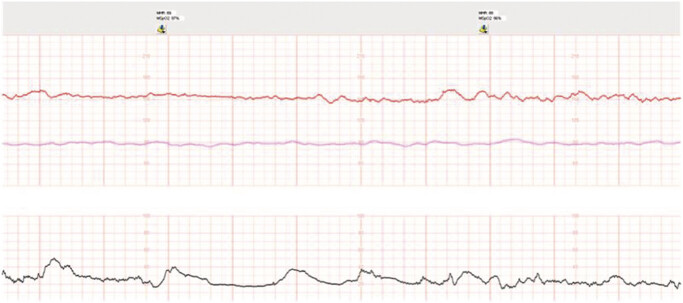
Hospital day 2 nonstress test.

On admission to the antepartum unit, a nonstress test (NST) was reassuring for gestational age with a baseline fetal heart rate of 160 beats per minute, moderate variability, accelerations, and no decelerations ( Fig. 1 ). She was initiated on the following pain control regimen: acetaminophen 1000 mg every 6 h, diphenhydramine 25 mg every 4 h, continuous fluids, and a hydromorphone patient-controlled analgesia (PCA) pump. Her pain significantly improved with these measures. An NST on hospital day 2 was also reassuring, with a baseline fetal heart rate of 150 beats per minute, moderate variability, accelerations, and no decelerations ( Fig. 2 ). The classical hematology service was consulted for further assistance in management of her pain crisis. The consulting team agreed with the primary team’s current management and recommended obtaining hemolysis markers, which returned notable for a lactate dehydrogenase of 832, a haptoglobin of <8, and a reticulocyte count of 21. Hematology also recommended a red blood cell transfusion if her pain did not improve by the following day. During this time, the patient developed a new-onset oxygen requirement (1 L nasal cannula), prompting a repeat chest X-ray, which was unremarkable.

**Fig. 3:**
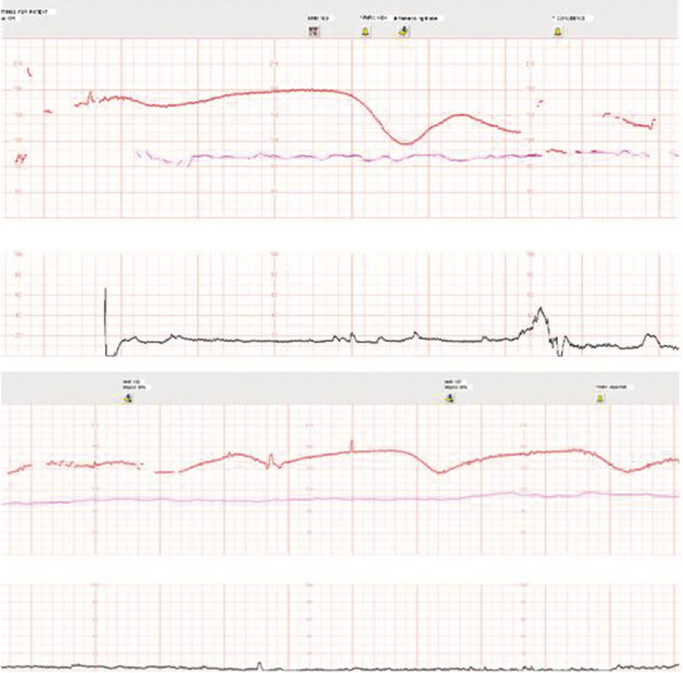
( **A** ) Hospital day 3 nonstress test, first 10 min. ( **B** ) Hospital day 3 nonstress test, second 10 min.

On the morning of hospital day 3, the patient was still reporting significant pain that had not improved since the day prior. Thus, the plan was made to transfuse 1 unit of packed red blood cells. She was also reporting decreased fetal movement, though this was not unexpected in the setting of a hydromorphone PCA. She was placed on the monitor for her daily NST, which demonstrated a Category 3 tracing with a tachycardic baseline of 175 beats per minute, absent variability, no accelerations, and recurrent spontaneous decelerations nadiring at 115 beats per minute ( Fig. 3A, B ). Thus, an emergent cesarean delivery was performed under general anesthesia.

A cesarean delivery with a high transverse hysterotomy was performed in the usual fashion. Intraoperative findings were remarkable for an extremely pale, hypoperfused uterus with almost no blood loss occurring at the time of hysterotomy. A limp, female neonate, weighing 1432 g, was delivered and immediately handed to the awaiting neonatology team. Profound uterine atony was encountered; however, minimal bleeding occurred. The atony was managed with methylergonovine, tranexamic acid, carboprost, misoprostol, and a B-Lynch suture. A preoperative hemoglobin level that was obtained while rolling to the operating room was notable for an acute drop to 6.0 g/dL. She was transfused with two units of packed red blood cells intraoperatively, and the total estimated blood loss for the procedure was 725 mL.

The infant was emergently resuscitated by the neonatology team. Umbilical cord arterial blood gas was remarkable for a pH of 6.99 and a base deficit of −14. The infant was completely unresponsive at the time of delivery, with no respirations or heart rate. Positive-pressure ventilation yielded no response, and intubation was performed. Asystole persisted, and chest compressions were initiated and continued until 12 min of life, when a heart rate was attained. APGARs were 0, 0, 0, 2, and 3 at 1, 5, 10, 15, and 20 min of life, respectively. Initial labs were remarkable for a hematocrit of 37%, an arterial pH of 6.75, a base deficit of −24, and a lactate of 15.6, consistent with severe hypoxic ischemic encephalopathy. Physical exam findings were remarkable for no movement, no respiratory effort, fixed pupils with no reactivity, and no reflexes. Pulseless electrical activity arrest then occurred, requiring a second round of cardiopulmonary resuscitation. After discussion with the parents, the decision was made to transition to comfort care. The infant was compassionately extubated in the patient’s arms.

The patient’s postoperative course was complicated by endometritis and an ongoing sickle cell crisis. A CTPE was obtained, given an ongoing oxygen requirement on postoperative day 2, which revealed mild pulmonary edema. She was able to be weaned off oxygen supplementation after diuresis with furosemide. She was restarted on hydroxyurea and required two more red blood cell transfusions. On postoperative day 4, she was discharged.

Placental pathology demonstrated prominent sickled maternal erythrocytes and increased fetal nucleated red blood cells. Infant autopsy was completed per parental request, which was remarkable for an extraordinarily friable brain and diffuse subarachnoid hemorrhages.

## Discussion

SCD in pregnancy is associated with substantial maternal and perinatal morbidity and mortality, including markedly increased risks of stillbirth and neonatal death. These risks are genotype-specific and appear consistently across geographic settings. A systematic review and meta-analysis reported a twofold increased risk in HbSC (RR: 1.87; 95% CI, 1.05–3.02) and a nearly fourfold increased risk of stillbirth (RR: 3.94; 95% CI, 2.60–5.96) and significant elevation in neonatal death risk (RR: 2.68; 95% CI, 1.49–4.82 in HbSS and *k* for HbSS). 1 A retrospective comparative cohort study in Africa demonstrated even higher relative risks for intrauterine fetal demise (RR: 12.00; 95% CI, 1.39–103.22) and early neonatal death (RR: 4.56; 95% CI, 1.09–19.10). 2 A multicountry meta-analysis similarly showed more than a fourfold increase in stillbirth (OR: 4.05; 95% CI, 2.59–6.32) and a near threefold increase in neonatal death (OR: 2.71; 95% CI, 1.41–5.22), with higher risks observed in high-income countries. 3

In this case, a previously stable gravida presented at 29 weeks of gestation with a vaso-occlusive crisis that rapidly led to acute fetal decompensation, resulting in the delivery of an infant with profound hypoxic ischemic encephalopathy who expired shortly after birth. The intraoperative surgical findings were notable for an extremely pale, hypoperfused uterus, and minimal surgical bleeding despite significant uterine atony, suggestive of a state of profound maternal and fetal circulatory compromise. Placental pathology demonstrating prominent sickled maternal erythrocytes and increased fetal nucleated red blood cells supports an antepartum period of impaired oxygen delivery.

While the patient underwent regular inpatient fetal surveillance during admission, nonreassuring fetal status was first detected on hospital day 3, prompting emergent delivery. Society of Maternal Fetal Medicine (SMFM) guidance for antenatal surveillance in uncomplicated SCD recommends weekly to twice-weekly testing beginning at 32–34 weeks, with earlier individualized testing for complicated cases. This patient’s acute deterioration occurred well before the standard initiation window, highlighting the difficulty of predicting sudden fetal compromise in SCD, even in the absence of preceding abnormal tracings.

The role of prophylactic transfusion in preventing such outcomes remains controversial. Historical protocols advocated for routine transfusion to reduce vaso-occlusive events, 5 6 but randomized controlled data from Koshy et al. found no improvement in perinatal outcomes despite fewer pain crises. 7 More recent evidence, however, suggests a potential benefit. A meta-analysis found that prophylactic transfusion was associated with reductions in perinatal mortality (OR: 0.43; 95% CI, 0.19–0.99) and neonatal death (OR: 0.26; 95% CI, 0.07–0.93), alongside decreases in maternal mortality, vaso-occlusive crises, and pulmonary complications. 8 In contrast, a more recent meta-analysis found that, while prophylactic transfusion may reduce the incidence of vaso-occlusive crisis (OR: 0.197; 95% CI, 0.08–0.49), it did not reduce rates of acute chest syndrome, venous thromboembolism, cesarean delivery, stillbirth, or neonatal death, underscoring the limitations of transfusion in preventing all adverse outcomes. It should be noted, however, that the low number of included studies resulted in a small sample size, possibly underpowering the analysis. 9

Guidelines reflect this mixed evidence. The American Society of Hematology (ASH) states there is insufficient evidence to recommend routine prophylactic transfusion for all pregnant patients with SCD, but it may be considered for women with a history of severe SCD-related complications or additional high-risk pregnancy features (e.g., other comorbidities, nephropathy) to reduce recurrent pain episodes, acute chest syndrome, or other complications. 10 The SMFM Consult Series No. 68 similarly recommends that prophylactic transfusion be individualized for high-risk patients, in accordance with ASH guidance, and undertaken through shared decision-making between hematology, maternal-fetal medicine, and the patient. 11

In this case, therapeutic transfusion (rather than prophylactic transfusion) was considered but not completed before fetal distress emerged, leaving unanswered speculation as to whether earlier initiation might have altered the course. Ultimately, this case underscores the ongoing risk of perinatal loss in SCD despite close monitoring and advanced obstetric care. It highlights the need for early multidisciplinary collaboration, careful risk stratification, and individualized consideration of prophylactic transfusion in high-risk pregnancies to optimize maternal and neonatal outcomes.
